# Paracrine HB-EGF signaling reduce enhanced contractile and energetic state of activated decidual fibroblasts by rebalancing SRF-MRTF-TCF transcriptional axis

**DOI:** 10.3389/fcell.2022.927631

**Published:** 2022-09-06

**Authors:** Junaid Afzal, Wenqiang Du, Ashkan Novin, Yamin Liu, Khadija Wali, Anarghya Murthy, Ashley Garen, Gunter Wagner, Kshitiz

**Affiliations:** ^1^ Division of Cardiology, Department of Medicine, University of California San Francisco, San Francisco, CA, United States; ^2^ Department of Biomedical Engineering, University of Connecticut Health, Farmington, CT, United States; ^3^ Department of Ecology and Evolution, Yale University West Campus, West Haven, CT, United States; ^4^ Systems Biology Institute, Yale University, West Haven, CT, United States

**Keywords:** contractility, energetics, cell-cell communication, cancer-stroma crosstalk, fetal maternal interface, placental invasion, paracrine HB-EGF signaling, SRF-Mrtf/Tcf axis

## Abstract

Multiple parallels exist between placentation and cancer dissemination at molecular, cellular, and anatomical levels, presenting placentation as a unique model to mechanistically understand the onset of cancer metastasis. In humans, interaction of placenta and the endometrium results eventually in deep invasion of placental extravillous trophoblasts (EVTs) into the maternal stroma, a process similar to stromal trespass by disseminating carcinoma cells. In anticipation of implantation, endometrial fibroblasts (ESFs) undergo a process called decidualization during the secretory phase of the menstrual cycle. Decidualization, among other substantial changes associated with ESF differentiation, also involves a component of fibroblast activation, and myofibroblast transformation. Here, using traction force microscopy, we show that increased cellular contractility in decidualized ESFs is reversed after interaction with EVTs. We also report here the large changes in energetic state of ESFs upon decidualization, showing increased oxidative phosphorylation, mitochondrial competency and ATP generation, as well as enhanced aerobic glycolysis, presenting mechanical contractility and energetic state as new functional hallmarks for decidualization. These energetic changes accompanying the marked increase in contractile force generation in decidualization were reduced in the presence of EVTs. We also show that increase in decidual contractility and mechanical resistance to invasion is achieved by SRF-MRTF transcriptional activation, achieved via increased phosphorylation of fibroblast-specific myosin light chain 9 (MYL9). EVT induced paracrine secretion of Heparin Binding Epidermal Growth Factor (HBEGF), a potent MAPK activator, which shifts the balance of SRF association away from MRTF based transcription, reducing decidual ESF contractility and mechanical resistance to placental invasion. Our results identify a new axis of intercellular communication in the placental bed modulating stromal force generation and resistance to invasion with concurrent downregulation of cellular energetics. These findings have important implications for implantation related disorders, as well as stromal control of cancer dissemination.

## Introduction

Parallels between placental invasion into the endometrium, and cancer metastasis has long been recognized (Costanzo et al., 2018; Coorens et al., 2021; Wagner et al., 2022). These parallels exist at multiple biological scales: genetic, molecular, cellular, and anatomical, and manifest in many similarities in the biochemical, immunological, or physical nature of interaction between cancer and stroma in one context, and placenta and endometrium in another (Suhail et al., 2021; Wagner et al., 2021). In humans, the endometrial fibroblasts (ESFs) undergo a cyclic pattern of differentiation, wherein ESFs differentiate into decidual ESFs (dESFs), resulting in increased cellular hypertrophy, transcriptional changes, increased secretion of hormones, and contractile force generation, in anticipation of implantation (Christian et al., 2002; Gellersen et al., 2007). For long, it was still not settled whether decidualization supports, or resists trophoblast invasion (Gleeson et al., 2001; Menkhorst et al., 2012; Pollheimer et al., 2018). In recent works, we have shown that decidualization significantly reduces trophoblast invasion, suggesting that the advent of dESFs is an evolutionary response to limit excessive invasion. We have also shown that decidualization as a process has evolved by incorporating a classical fibroblast activation response to wounding (Wu et al., 2020), which occurs in response to the degradation of uterine epithelium by trophoblasts, and their invasion into the maternal stroma.

Fibroblast activation generically results in increased actomyosin assembly and force generation, followed by remodeling of the extracellular matrix. Contractile force generation by fibroblasts, when coupled with fibroblast-epithelium interaction, is now understood to be important in instigating dissemination of epithelial tumors (Labernadie et al., 2017; Ansardamavandi and Tafazzoli-Shadpour, 2021). However, contractile forces may also be resistive (Wang et al., 2021). Drawing parallels between cancer-fibroblast interaction, and trophoblast-ESF interaction (Kshitiz et al., 2019), we asked how the decidual stromal contractile force generation change after interaction with trophoblasts. Generation of contractile force is an energy intensive phenomenon. We found that decidualization is accompanied by a marked increase in the energetic state, increasing both oxidative phosphorylation (OxPhos) and glycolysis. We also found that the EVT induced reduction in contractile force generation is also accompanied by correlated reduction in glycolysis, as well as oxidative phosphorylation.

We have also identified a cell-cell paracrine interaction axis between EVTs and dESFs, which mediated the reversal in dESF contractile force generation, and increased invasability. Specifically, we found that secretion of HB-EGF (heparin bound-endocrine growth factor) by HTR8 resulted in rewiring of SRF (serum response factor) association from MRTF (myocardin related transcription factor) to TCF (ternary complex factor). Reduced SRF-MRTF transcriptional regulation reduces phosphorylation of myosin light chain 9 (MYL9) resulting in decreased contractile force generation in dESFs. HB-EGF is an abundantly secreted growth factor in many cancers (Sethuraman et al., 2018), but its role in regulating stromal contractility, and invasability has not been studied. Our study underlines that fibroblast mechanics and energetic state are crucial in the stromal containment of epithelial invasion, and that both invasive cells, here trophoblasts, can evade the mechanical resistance offered by stromal fibroblasts by paracrine modulation of their contractile machinery.

## Results

### Extravillous trophoblasts reduce mechanical force generation by decidual endometrial fibroblasts

**FIGURE 1:**
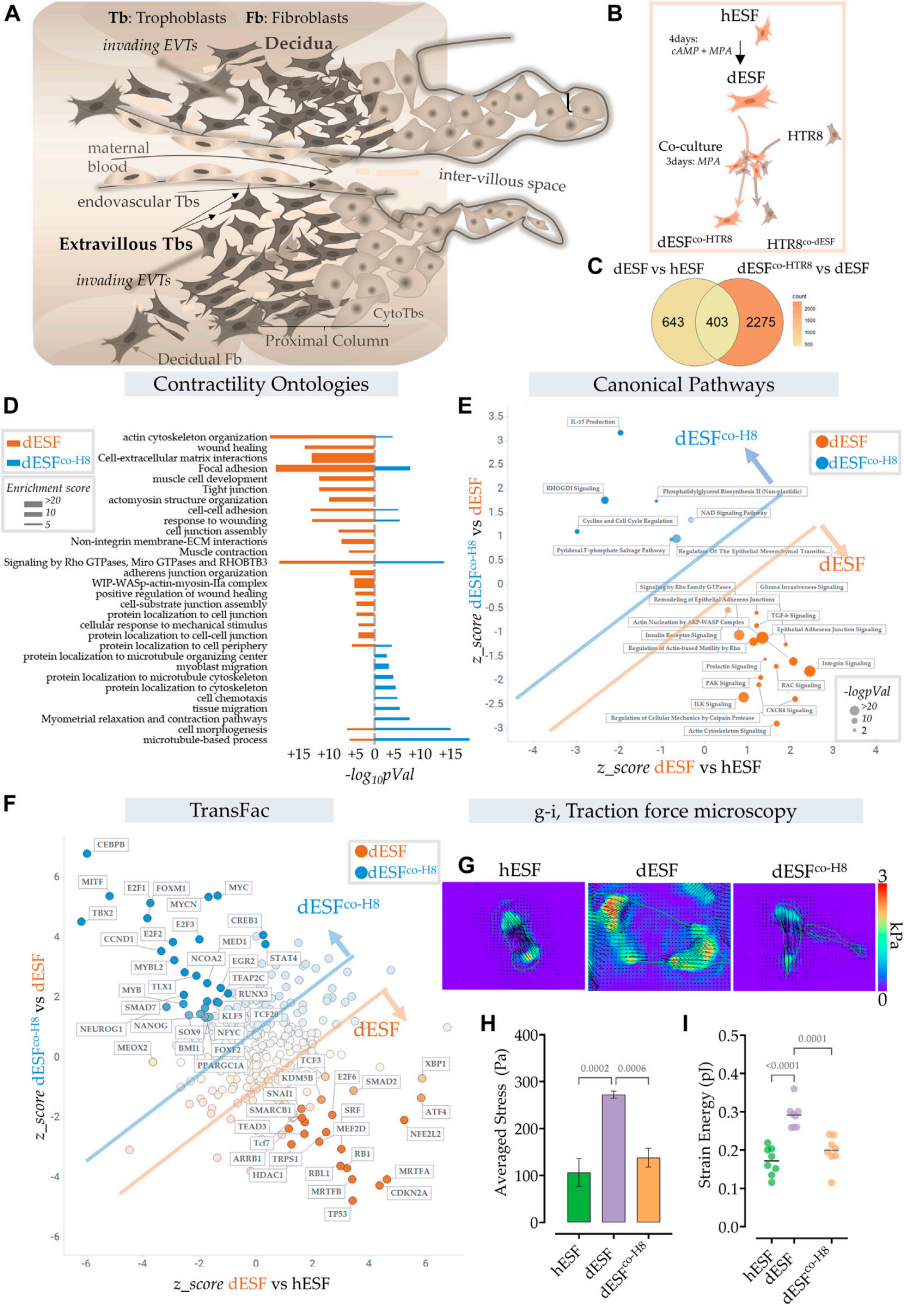
Extravillous trophoblasts reduce contractile force generation by decidual endometrial fibroblasts. **(A)** Schematic showing the maternal fetal interface during placentation, wherein EVTs escape into the maternal stroma and deeply invade into the endometrium to eventually reach the spiral arteries. **(B)** Schematic showing the experimental plan for co-culture of dESFs with fluorescently labeled HTR8; cells were separated by fluorescent assisted cell sorting after 3 days of co-culture and sequenced. **(C)** Venn diagram showing gene counts differentially regulated between undifferentiated ESFs (hESFs), and decidualized ESFs (dESFs), and between dESFs co-cultured with HTR8 (dESFco-H8) and dESFs. **(D)** Activation of Contractility related ontologies in dESFs and dESF^co−H8^. Thickness of bar plot shows enrichment score of the relevant ontology. **(E)**. Activation of canonical pathways in dESFs compared to hESFs or in dESFco-H8 vs. dESFs; Axes represent the activation or inhibition (z-score) of relevant ontologies; Ontologies that are upregulated in dESFs (compared to hESFs) but go down with co-culture (dESF^co−H8^) are labeled orange, and ontologies upregulated in dESF^co−H8^ are labeled blue. Size of the bubble reflect -log (p-Val) in that quadrant. Calculated using Ingenuity Pathway (IPA) analysis. **(F).** Transcriptional factors (TransFac) upregulated in dESFs and then downregulated after co-culture are labeled orange) or downregulated with decidualization (dESFs), and then upregulated with co-culture (dESF^co−H8^) are labeled blue. Calculated using Ingenuity Pathway (IPA) analysis. **(H–I)**. Traction force microscopy: **(H)**. Representative traction force vector maps and strain energy maps in hESF with decidualization (dESF) and after co-culture treatment (dESFco-H8). Anova with Tukey’s correction was used for quantification of average cellular stress **(H)**, and strain energy **(I)**; *n* > 10 cells per condition.

Extravillous trophoblasts (EVTs), which are notably of recent evolutionary origin (Carter, 2012, 2021), invade through the decidual stroma, to reach the maternal spiral arteries. In the process, EVTs interact with the decidual endometrial fibroblasts (dESFs) (Figure 1A). We have previously reported that decidualization is evolutionarily derived from fibroblast activation (Wu et al., 2020). Indeed, we had found that differentiation of ESFs involves an interim stage which mimics activation of fibroblasts in response to wounding, which gets extended to a larger program of decidualization (Wu et al., 2020). Early decidualization, therefore, appeared to be mimicking increase in ESF contractility and generation of mechanical forces. We therefore asked if interaction of dESFs with EVTs may influence dESF mechanical force generation. We have previously reported that EVTs could modulate the matricellular homeostasis achieved by decidualization, which directly reverses the decidual-specific resistance to placental invasion. Here, we focused on fibroblast force generation, and associated fibroblast metabolism to ask if interaction with EVTs may modulate the force generation capability of decidualized ESFs.

We used RNAseq data obtained from ESFs decidualized for 4 days and co-cultured for 2 days with fluorescently labeled HTR8, an EVT cell line. After incubation, ESFs and EVTs were seperated using fluorescence assisted cell sorting (FACS) (Figure 1B). Co-culture with HTR8 resulted in significant change in gene expression of dESFs, with many genes that were reversed in expression after co-culture (Figure 1C). Focusing on biomechanical pathways associated with fibroblast activation, we found that overall there was a significant effect of HTR8 co-culture on dESF gene expression (Figure 1D).

To confirm whether co-culture induced reduction in dESF gene expression related to biomechanical force was systemic, we computed activation of canonical pathways of contractile force generation in decidualization, and after co-culture with HTR8 (Figure 1E). We found that many gene ontologies related to cellular contractile force generation, including TGFβ signaling, actin nucleation, Rho mediated action-based motility, Rac signaling, and Calpain protease mediated cellular mechanics were upregulated in dESFs vs. hESFs. Their activation were markedly reduced after co-culture with HTR8 in dESF^co−H8^ (Figure 1E). Transfac based Transcription Factor activation scoring also showed that key transcriptional regulators associated with cellular motility, contractile force generation, and fibroblast activation were initially upregulated in dESFs, and showed reversal when dESFs were in co-culture with HTR8. These included myocardin related TFs, MRTF-A/B, and serum response factor (SRF), which together can form a transcriptional factor complex activating expression of many genes mediating cellular force generation (Gualdrini et al., 2016). Others included SNAI1, a key factor regulating epithelial to mesenchymal transformation, as well as MEF2D encoding myocyte enhancer factor 2D, a key TF regulating genes encoding myosin based contractile machinery.

To functionally confirm that co-culture with HTR8 had indeed resulted in reduction in force generation by dESFs, we used traction force microscopy, which computes contractile forces generated by adhered cells from displacement of fluorescent beads embedded in a pliable hydrogel based cell substratum. dESFs were cultured, either after decidualization, or conditioned with supplemented medium from HTR8. We found that contractile force generation in dESFs was markedly higher than in hESFs, and treatment with conditioned medium from HTR8 reduced it significantly (Figures 1G,H). Strain energy density, normalized to cell area also showed a similar trend of an initial increase, and then decrease after co-culture (Figure 1I). Reduction in the contractile force generated by dESFs by conditioned medium suggested involvement of paracrine signals from HTR8 in regulating fibroblast mechanics.

### Decidualization enhances oxidative phosphorylation and mitochondrial ATP generation in ESFs, which is reduced after co-culture with EVTs

**FIGURE 2:**
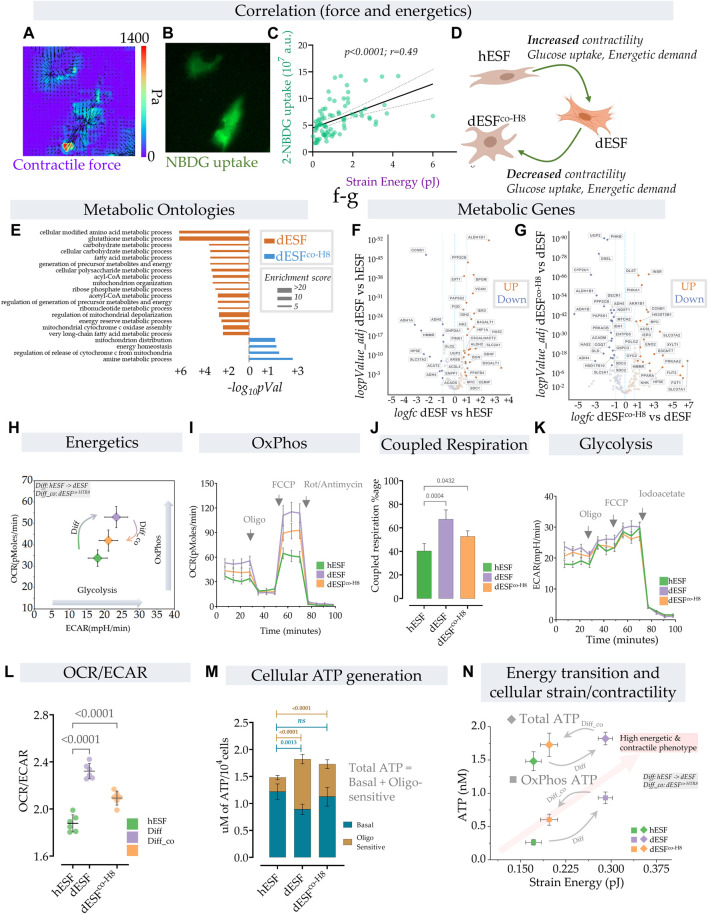
Energetic transition in decidual ESF reflect changes in contractility which is reversed by EVT. **(A–D)** Traction force microscopy and glucose uptake: **(A)** Glucose uptake was monitored within a population of decidualized ESFs (dESF) with NBDG along with cellular contractility, Representative figure of two contracting cells with **(A)** traction force vector maps and **(B)** NBDG fluorescence **(B)**; **(C)** Correlation of strain energy of contracting cells and glucose uptake; *n* > 70. **(D)** Schematic showing increased contractility, glucose uptake, and energetic demand in decidualized cells (dESF) that is reduced with co-culture (dESF^co−H8^). **(E)** Metabolic ontologies that are activated in dESF or dESF^co−H8^ with their enrichment score **(F,G)**. Volcano plot of differential metabolic genes in combined ontologies of ATP generation, Oxidative phosphorylation, and glycolysis: Significant number of metabolic genes are upregulated (highlighted genes are < *p*0.001) with differentiation and several key TCA (citric acid cycle) genes are downregulated after co-culture with hTR8. **(H,I)** Energetics data in hESF, decidualized hESFs (dESF) and co-culture condition (dESFco-H8) using XF analyzer. **(H)** Basal oxygen consumption rate (OCR) and extracellular acidification rate (ECAR) shows an increase in OxPhos and glycolysis with decidualization which is partially reversed with co-culture condition. Basal data is presented with mean and SD. **(I)** Increase in oxygen consumption rate (OCR) at basal levels, enhanced coupled (oligomycin sensitive) and uncoupled respiration (FCCP) with decidualization. **(J)** Percentage of coupled respiration: oligomycin sensitive respiration is significantly increased in dESFs compared to hESF and dESF^co−H8^. **(K)** Extracellular acidification rate: Increase in ECAR with decidualization reflects increase in glycolysis which is confirmed after inhibition with iodoacetate. *n* = 6 in each XF experiment with data reported as mean ± SD and statistical significance calculated using Anova followed by Dunnett’s test. **(L)** OCR/ECAR ratio at basal levels reflects increased reliance on oxygen consumption in dESFs. *n* = 6 in each XF experiment with data reported as mean ± SD and statistical significance calculated using Anova followed by Dunnett’s test. **(M)** Increase in total cellular ATP generation with decidualization. Oligomycin sensitive ATP generation which reflects ATP generation from OxPhos indicates increased utilization of oxidative phosphorylation for ATP generation in dESFs. *n* = 6 in ATP experiment and the data is presented as mean ± SD. Statistical significance was calculated using Anova followed by Šídák’s test. **(N)** Transition of cellular contractility (strain energy) and ATP levels per cell (total and Oxphos) show high energetic and contractile phenotype with decidualization.

Large contractile force generation is a highly energetically intensive process, necessitating rewiring of the metabolic flux in a cell. As decidualization involves an early onset of fibroblast activation, accompanied by increase in mechanical force generation (Figures 2A–C), we expected an increase in energy demand in the cell (Sleep et al., 2005). To functionally test if glucose uptake and force generation are linked, we took advantage of the inherent diversity in strain energy in a population of dESFs, and correlatively measured NBDG (glucose) uptake and contractile force generation (Figures 1A,B). We found a high correlation between NBDG uptake and strain energy within dESFs (Figure 2C), confirming that increased contractile force generation poses higher energy demands on the cell (Figure 2D). How decidualization affects metabolism and energy requirement in ESFs has not been well studied, and there is little understanding of the energetic transitions in dESFs as they interact with EVTs during placentation. We therefore systematically tested the energetic state of ESFs in response to decidualization, as well as subsequent co-culture with HTR8s.

Although enhanced glucose uptake suggested increase in aerobic glycolysis, gene set analysis for metabolic ontologies showed that decidualization also resulted in enrichment of several ontologies related to mitochondrial respiration (Figure 2E). Key metabolic genes and mitochondrial transcripts that regulate citric acid cycle (TCA) and ATP generation through mitochondria were also increased with decidualization (Figures 2F,G). Increase in mitochondrial ATP generation is an efficient process whereby ATP generation per glucose molecule is significantly increased from 2–4 mol/glucose molecule to 32–36 mol of ATP. Increase in ATP however limits cellular substrate (glucose) uptake (Blodgett et al., 2007). To keep the high glucose levels for proliferative or other needs, cells have adaptive mechanisms to increase the pyruvate to lactate flux, limit OxPhos and/or increase ATP utilization (Melo et al., 1998; Hardie, 2000; Garcia-Cao et al., 2012).

In order to understand the parallel increase in glucose uptake, as well as several key mitochondrial energetics transcripts, we performed a comprehensive energetic measurement in decidualized ESFs, as well as after subsequent treatment with HTR8 conditioned medium. Using XF analyzer to simultaneously measure oxidative phosphorylation (OxPhos) and glycolysis, we asked whether the increase in energy substrate uptake with concomitant increase in cellular contractility also leads to a parallel increase in aerobic glycolysis (Figure 2H). Surprisingly, we observed an increase in both Oxphos and aerobic glycolysis with decidualization. Both the basal mitochondrial respiration rate and extracellular acidification rate (ECAR), indicative of OxPhos and aerobic glycolysis respectively, were increased in decidualization, suggesting the transition of cells towards high energy state (Figure 2H). Interestingly, treatment with HTR8 conditioned medium resulted in a decrease of both Oxphos and aerobic glycosis (Figure 2H).

Furthermore, decidualization not only resulted in increased basal respiration but also caused a significant increase in coupled respiration (with ATP synthase inhibitor oligomycin) and maximum respiratory chain capacity (FCCP) (Figure 2I). The increase in respiration that was coupled to ATP generation (coupled respiration) was close to ∼67% in decidualized cells compared to ∼40% in hESFs, and ∼52% with co-culture condition (Figures 2I,J). These results indicate not only increase in mitochondrial respiration, but also increase in mitochondrial efficiency with decidualization. Extracellular acidification rate (ECAR) was also increased with decidualization (Figure 2K). Although increase in both OxPhos and aerobic glycolysis suggest high energetic state of cells in decidualized stage, the OCR/ECAR ratio which is independent of cell number indicated preferential increase in mitochondrial respiration in decidualized cells (Figure 2L). Interestingly, although the total ATP levels were increased with decidualization from ∼1.47 nM/cell to ∼1.72 nM, a significant increase in ATP generation was observed through mitochondria which were sensitive to oligomycin (Figure 2M).

These results show that decidualization involves large changes in the energetic state of fibroblasts, both in ATP generation within the mitochondria, as well as large increase in carbon flux, likely for anabolic processes. Oxphos is increased typically as cells differentiate and leave the undifferentiated proliferative state (Zheng et al., 2016). However, the large increase in ATP generation in dESFs suggest that Oxphos produced ATP likely primarily fuels large increase in contractile force generation. HTR8s cause a significant reversal in both these fundamental changes in the energetic state of dESFs. Indeed, increase in glucose flux after decidualization does not result in a significant contribution towards ATP generation through glycolysis. The increase in cellular energy demand (ATP) with decidualization is primarily met with an increase in energy efficient OxPhos pathway. Significantly high ATP generation in dESFs vs. hESFs, also more proportionately met by Oxphos, indicate the metabolic flux meeting the needs for the marked increase in contractile force generation (Figure 2M). Correlating the strain energy, and ATP generation showed that increased mechanical strain energy generation in response to decidualization also results in large increase in ATP generation in the mitochondria (OxPhos), and treatment with conditioned medium from HTR8 restored the low ATP generation energetic state in the mitochondria, correlatively with reduced strain energy (Figure 2N).

Overall, increase in both glucose uptake and mitochondrial respiration capacity/mitochondrial ATP generation indicates an increase in ATP demand that is met by OxPhos and a possible diversion of glucose carbon towards biosynthetic needs as indicated in upregulation of several metabolic ontologies, transcripts and increase ECM/collagen production with decidualization (Figure 2N). The concomitant increase in glycolysis and activation of several biosynthetic gene ontologies with decidualization indicate a shift in metabolic pathways towards biosynthetic processes (Vander Heiden et al., 2009), which was partially reversed by interaction with EVTs.

### EVTs reverse decidualization induced increase in activation of MRTF transcriptional regulation

**FIGURE 3:**
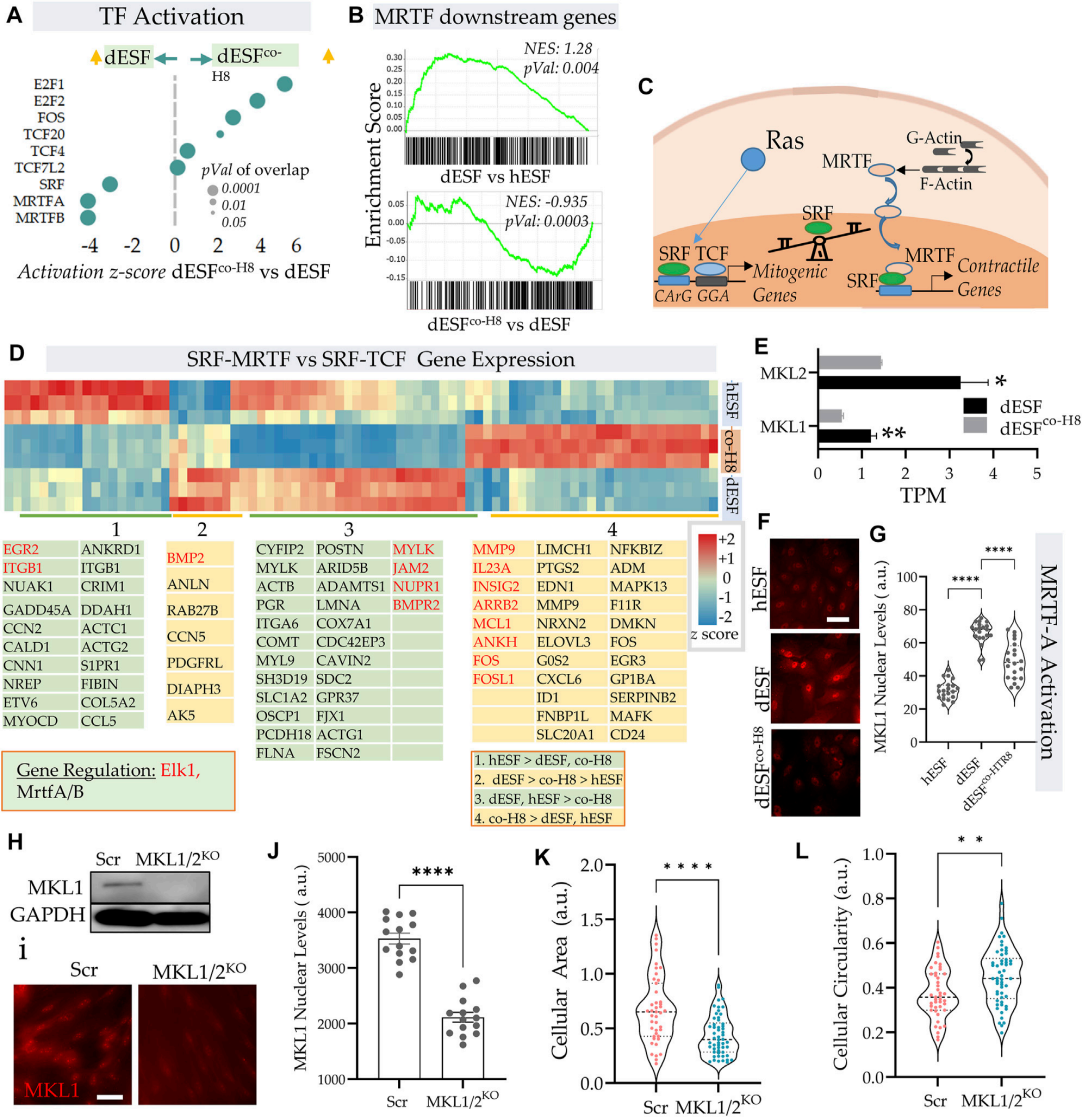
EVTs reverse MRTF activation in decidual fibroblasts. **(A)** Predicted IPA based activation scores of top transcription factors which are reduced in dESFs co-cultured with HTR8 feature MRTF-A, and MRTF-B, as well as SRF. Bubble size reflects *p*-value overlap of genes. **(B)** Gene set enrichment analysis (GSEA) of SRF-MRTF downstream genes in dESF vs. hESF (top), and dESF^co−H8^ vs. dESF (bottom); Enrichment score, and *p*-value shown in inset; leading edge genes for both comparisons shown at the bottom. **(C)** Schematic showing a balance of contractile and mitogen phenotype maintained by association of MRTA or TCF co-factors with SRF. **(D)** Heatmap showing z-scores genes activated by SRF in hESF, dESF and dESF^co−H8^. Hierarchical clustering by Euclidian distance revealed 4 main clusters that were either upregulated in hESF, dESF, both hESF and dESF, or in co-culture condition. Genes that are downstream of Elk1 activation are coded with red color and MRTFA/B are coded with black color. Color intensity bar shows z score calculated from TPM values. **(E)** Transcripts per million (TPM) for MKL1 and MKL2 in dESF, and dESF^co−H8^; n = 3 samples. **(F)** Representative immunofluorescence images showing MKL1 (MRTF-A) expression in hESF, dESFs, and dESF^co−H8^, with quantification of fluorescence levels in the nuclei shown in **(G)**; significance was calculated using Anova followed by Tukey’s test; *n* = 20 for each condition. **(H)** Immunoblot showing reduced MKL1 abundance in dESFs before, and after silencing of both MKL1 and MKL2 genes (MKL1/2), lower lane shows abundance of GAPDH; also confirmed with immunofluorescence **(I,J)**. **(K,L)** Morphological changes in dESFs in response to MKL1 and MKL2 gene silencing in dESFs, showing single cell area **(K)**, and cellular circularity **(L)**.

IPA pathway analysis revealed an increased activation of serum response factor (SRF) and myocardin-related transcription factors (MRTFs) (Figure 1F). Indeed, both MRTF-A and MRTF-B, as well as SRF were among the top transcription factors that were predicted to be activated with decidualization, and then decreased after EVT co-culture (Figure 3A). GSEA analysis revealed a significant enrichment of MRTF downstream genes in dESF vs. hESF, which was also reduced in dESF^co−H8^ (Figure 3B). MRTFs in association with SRF are key TFs regulating muscle machinery adaptation to workload (Montel et al., 2019), and along with YAP-TAZ, impart contractile properties to cancer associated fibroblasts (Foster et al., 2017). Activation of SRF-MRTF, as well as SRF independent MRTF activation are important mediators of mechanical forces essential for cancer cell migration through dense extracellular matrix (Gau and Roy, 2018). MRTFs competitively associate with serum response factor (SRF) against TCFs (Erk regulated ternary complex factors), in response to actin polymerization. Upon actin polymerization, MRTFs can localize in the nucleus, and bind to SRF to activate gene transcription of several genes regulating cellular contractility and force generation (Gualdrini et al., 2016; Gau and Roy, 2018) (Figure 3C). Indeed, many genes downstream of MRTFs showed increased expression with decidualization, while genes downstream of TCF factors were preferentially expressed after co-culture (Figure 3D). RNAseq showed significant reduction in the transcripts levels of both MKL1 and MKL2 (Figure 3E), while immunofluorescence showed that MRTF-A (MKL1) localization was primarily nuclear in dESFs compared to hESFs, and treatment with conditioned medium from HTR8 reversed the trend (Figures 3F,G), corroborating the predicted transcriptional activation of SRF-MRTF signaling in dESFs and its reduction after EVT interaction. CRISPR Cas9 mediated gene silencing of both MKL1 and MKL2 showed reduced MKL1 abundance and nuclear localization (Figures 3H–J). Gene silencing for MKL1 and MKL2 also resulted in significant reduction in cell area (Figure 3K), while cellular circularity which negatively correlates with the elongated spindle shape formation was significantly increased (Figure 3L). Overall, gene expression data indicated increased SRF-MRTF mediated transcriptional activation in decidual cells, which was reversed after interaction with EVTs.

### MRTFs regulate mechanical resistance in decidual fibroblasts to trophoblast invasion

**FIGURE 4:**
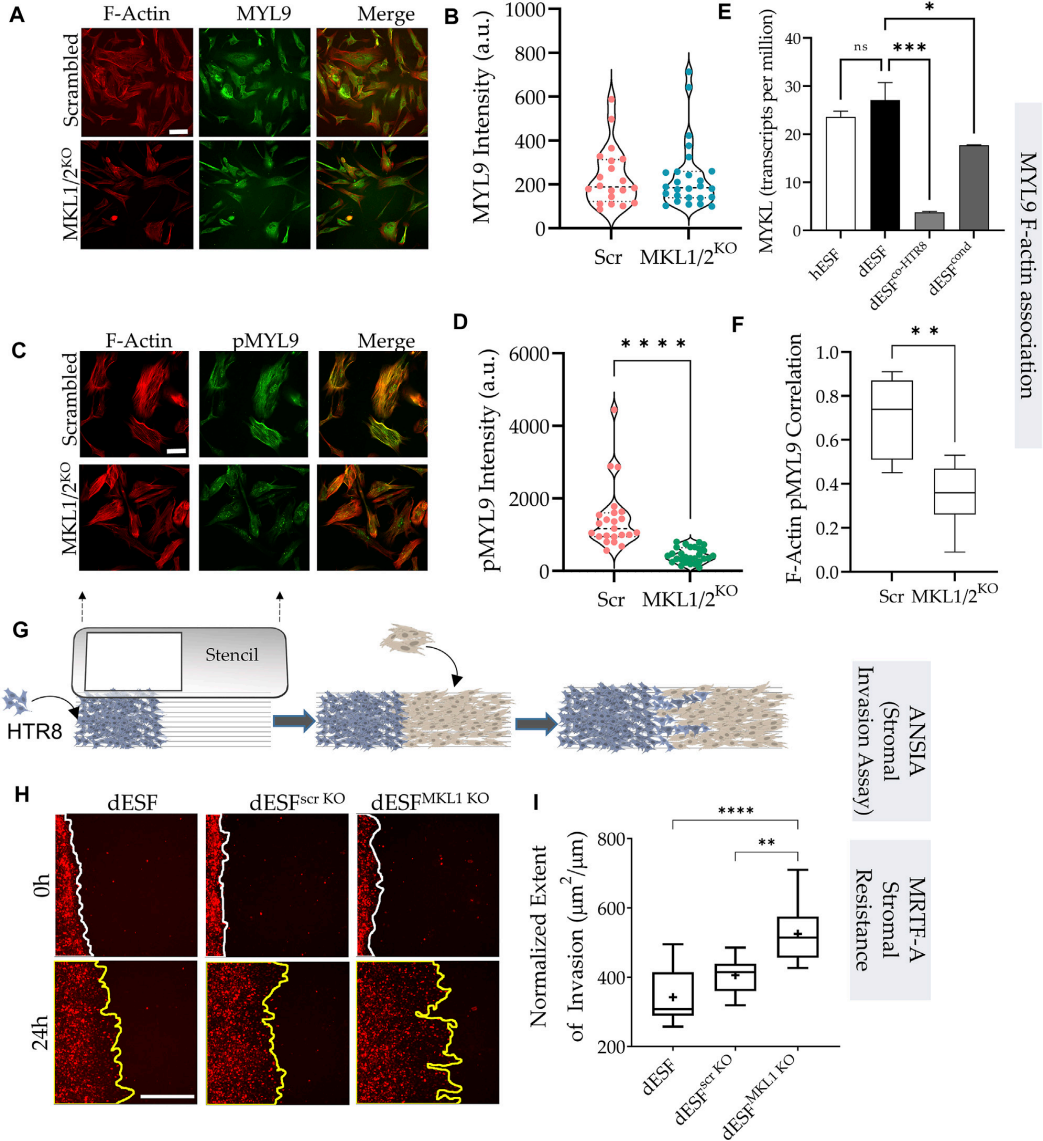
MRTF silencing in decidual fibroblasts reduces actomyosin contractility and resistance to trophoblast invasion. **(A)** Immunofluorescence of dESFs with CRISPR/Cas9 based gene silencing for scrambled control, and MKL1 & MKL2 (MKL1/2^KO^) showing F-actin and myosin light chain-9 (MYL9), quantification of intensity per cell for MYL9 shows no statistical difference **(B)**; scale bar = 25 µm. **(C)** Immunofluorescence images showing reduced abundance of phospho-MYL9 in MKL1/2^KO^ dESFs **(D)**; scale bar = 20 µm. **(E)** Relative expression of myosin light chain kinase (MYLK) hESF, dESF, as well as dESFs co-cultured or conditioned with HTR8. **(F)** Pearson’s coefficient of phospho-MYL9 (green) co-localization with F-actin fibers (red) in Scrambled control, and MKL1/2^KO^ dESFs. **(G)** Schematic showing the setting of the ANSIA assay to measure stromal invasion of H2B-mCherry expressing HTR8s in the dESF monolayer on a nanopatterned substrate with the anisotropic nanoridges aligned orthogonal to the patterned HTR8-dESF interface. **(H)** Representative time-stamped images of HTR8 (red)-dESF (unlabeled) interface at 0 and 24 h, with dESFs either silenced with scrambled gRNA, or gRNA targeted towards MKL1. **(I)** Extent of stromal invasion by H2B-mCherry labeled HTR8 cells into the dESF monolayer for conditions in e, and significance is calculated using Anova followed by Tukey’s test; *n* > 10 per condition.

Traction force microscopy showed that silencing both MRTF-A (encoding MKL1) and MRTF-B (encoding MKL2) genes resulted in significant reduction in strain energy density (Figures 4A,B). We sought to identify how MRTFs may regulate actomyosin contractility at a molecular level. Upon being phosphorylated, myosin light chain homologues engage with actin filaments, generating contractile forces in cells. MYL9 is the chief homologue of myosin light chain encoding genes within fibroblasts, and has been described to be transcriptionally regulated by MRTFs. However, immunostaining for MYL9 did not show much difference between control dESFs, and those silenced for MKL1 and MKL2 genes (Figures 4A,B). Immunofluorescence also showed expectedly that MYL9 did not colocalize with F-actin fibers. However, phosphorylated MYL9 (pMYL9) was significantly lower in dESFs with MKL1 and MKL2 silenced (MKL1/2^KO^) (Figures 4C,D). Notably, myosin light chain kinase (MYLK) is also transcriptionally expressed by MRTFs (Johnson et al., 2014 #67), and was significantly reduced in dESFs after co-culture of conditioning with HTR8 (Figure 4E), suggesting that MRTFs may increase phosphorylated MYL9 in dESFs. Colocalization analysis also showed that pMYL9 was associated strongly with F-actin fibers, and this association reduced significantly in MKL1/2^KO^ cells (Figure 4F). Overall, these data indicated that MRTFs may be regulating dESF contractile forces via increased phosphorylation of MYL9, likely by transcription of MYLK.

Could the increased contractile force generation likely by downstream targets regulated by MRTFs contribute to increased decidual resistance to trophoblast invasion? Although, contractile force generation by cancer associated fibroblasts has been shown to be critical in inducing dissemination of epithelial cancer (Ansardamavandi and Tafazzoli-Shadpour, 2021), the actual response of collective fibroblast activation in a wound, or in response to decidualization, in resisting epithelial invasion is not well understood. Both epithelial-fibroblast coupling via N- and E-Cadherin heterotypic adhesion (Labernadie et al., 2017), as well as the collective directionality of force presentation against the invading front because of remodeled matrix may contribute to the process of stromal resistance (Erdogan and Webb, 2017; Asif et al., 2021).

Harnessing the large differences in comparative placentation across mammals, we have demonstrated that stromal invasion is both an outcome of the invading cells, as well as the active resistance, or assistance, offered by the stromal fibroblasts (Kshitiz et al., 2019). Indeed, across mammals, the large differences in placental invasion is primarily an outcome of differential stromal invasability, a selected and genetically regulated phenotype (Kshitiz et al., 2019; Suhail et al., 2019). We have shown that decidualization results in significantly high resistance to invasion by trophoblasts, also borne from the observation that ectopic pregnancies in non-decidualized regions of the uterus are highly invasive (Randall et al., 1987). We therefore asked whether decreased MRTF activation in response to EVT co-culture may also regulate decidual invasability.

Towards this objective, we used a quantitative and high sensitivity assay to measure stromal invasability which we have previously described, called Accelerated Nanopatterned Stromal Invasion Assay (ANSIA) (Novin et al., 2021). As stromal invasion is a very slow phenotype to observe at cellular and subcellular resolution, we have used a collagen matrix mimicking anisotropic pattern to align the actomyosin assemblies in individual cells in a single direction (Figure 4G). H2B-mCherry labeled HTR8 and unlabeled dESFs are patterned using a stencil to create an interface orthogonal to the direction of the underlying nanoridges (Figure 4G). Before plating, dESFs were either left untreated, or silenced with scrambled control, or gRNA targeted against MKL1 gene. HTR8 invasion was observed for 24 h using fluorescence live microscopy, and invasive spread measured and normalized to the initial interface length between HTR8 and dESFs. We found that MKL1 gene knockout significantly decreased the resistance offered by dESFs to HTR8 invasion, with HTR8 forging deep collective fronts into the stroma (Figures 4H,I). These data showed that co-culture with HTR8 resulted in decreased SRF-MRTF downstream gene expression, and that MRTF regulates stromal actomyosin force generation and resistance to trophoblast invasion.

### HB-EGF secreted by HTR8 rewire SRF association with MRTFs in decidual fibroblasts

The effect of EVTs on reduction of dESF contractility was maintained both in co-culture, as well as from conditioned medium from HTR8, suggesting that paracrine signals from HTR8 may play a role in regulating dESF contractility. Because SRF-MRTF signaling was predicted to be highly active in dESF, and was also reduced significantly by HTR8 conditioned medium (Figure 3B), we sought to identify secreted factors that could potentially regulate MRTF activation.

**FIGURE 5:**
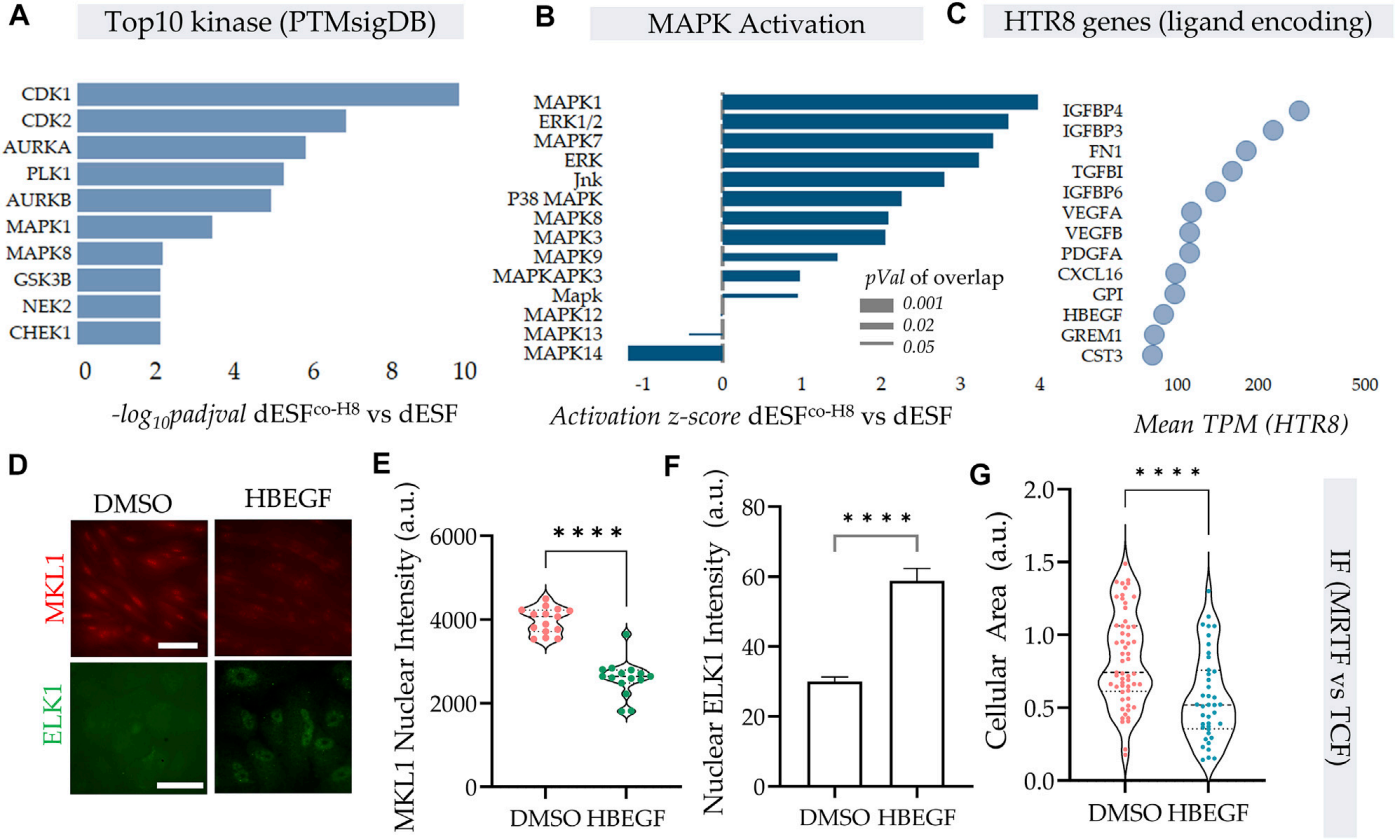
HB-EGF secreted from EVTs shift SRF-MRTF to SRF-TCF transcription. **(A–C)** HTR8 secreted HB-EGF activate MAPK signaling in dESF. **(A)** Top 10 kinases that are activated with HTR8 co-culture (dESF^co−H8^ vs. dESF) based on differential genes that are downstream of the predicted kinases, evaluated using PTM signatures database; Data presented with FDR correction of pVal. **(B)** IPA predicted activation score of MAPK species in dESFco-H8 vs. dESFs. **(C)** Bubble plot showing TPM values of genes encoding ligands expressed in HTR8. **(D)** Immunofluorescence images showing MKL1 (encoded by *MRTFA*) and ELK1 (component of TCF complex) localization in dESF without, or with treatment with recombinant HB-EGF, with quantification for MKL1 nuclear levels shown in **(E)**, and Elk1 cytoplasmic and nuclear intensities shown in **(F,G)**.

Protein kinases are essential in regulation of cellular contractility and motility, and RNA seq data indicated that MAPK were top kinases activated on PTMsigDB (Figure 5A). Further, several of the MAPK were also putatively activated as predicted by IPA analysis (Figure 5B) in decidualized cells (Krug et al., 2019; Kuleshov et al., 2021). As described earlier, TCF components, which are Erk activated act as antagonists to SRF-MRTF signaling, by competitive binding to SRF in the nucleus (Gau and Roy, 2018). We therefore sought to identify paracrine sources from HTR8 which could activate MAPK signaling in dESF, and potentially reduce SRF-MRTF signaling. Using RNAseq of HTR8 we identified the genes encoding secreted ligands which may be responsible for modulating MRTF signaling (Figure 5C). We identified heparin bound epidermal growth factor (HB-EGF), a key enzyme implicated in implantation and placentation, and a potent activator of Erk signaling, which could regulate TCF localization in the nucleus antagonizing SRF-MRTF interaction (Figure 5C). As gene enrichment analysis had revealed a marked activation of MAPK pathway in dESFs after HTR8 co-culture (Figure 5B), it laid credence to the hypothesis that intercellular signaling through HB-EGF may modulate intracellular dESF signaling. We therefore tested if HB-EGF could indeed alter MRTF signaling in dESFs. Immunofluorescence revealed that treatment of dESFs with recombinant human HB-EGF resulted in a marked decrease in the nuclear localization of MKL1 (MRTF-A), as well as concomitantly, increased nuclear localization of Elk1, suggesting that HB-EGF is capable of tipping the balance in dESF from SRF-MRTF to SRF-TCF signaling (Figures 5D–F). Additionally, HB-EGF treatment also reduced cell size (Figure 5G), similar to what was observed in MRTF gene silencing (Figure 3K).

### HB-EGF reduces actomyosin contractility and stromal resistance acquired in decidual fibroblasts

**FIGURE 6:**
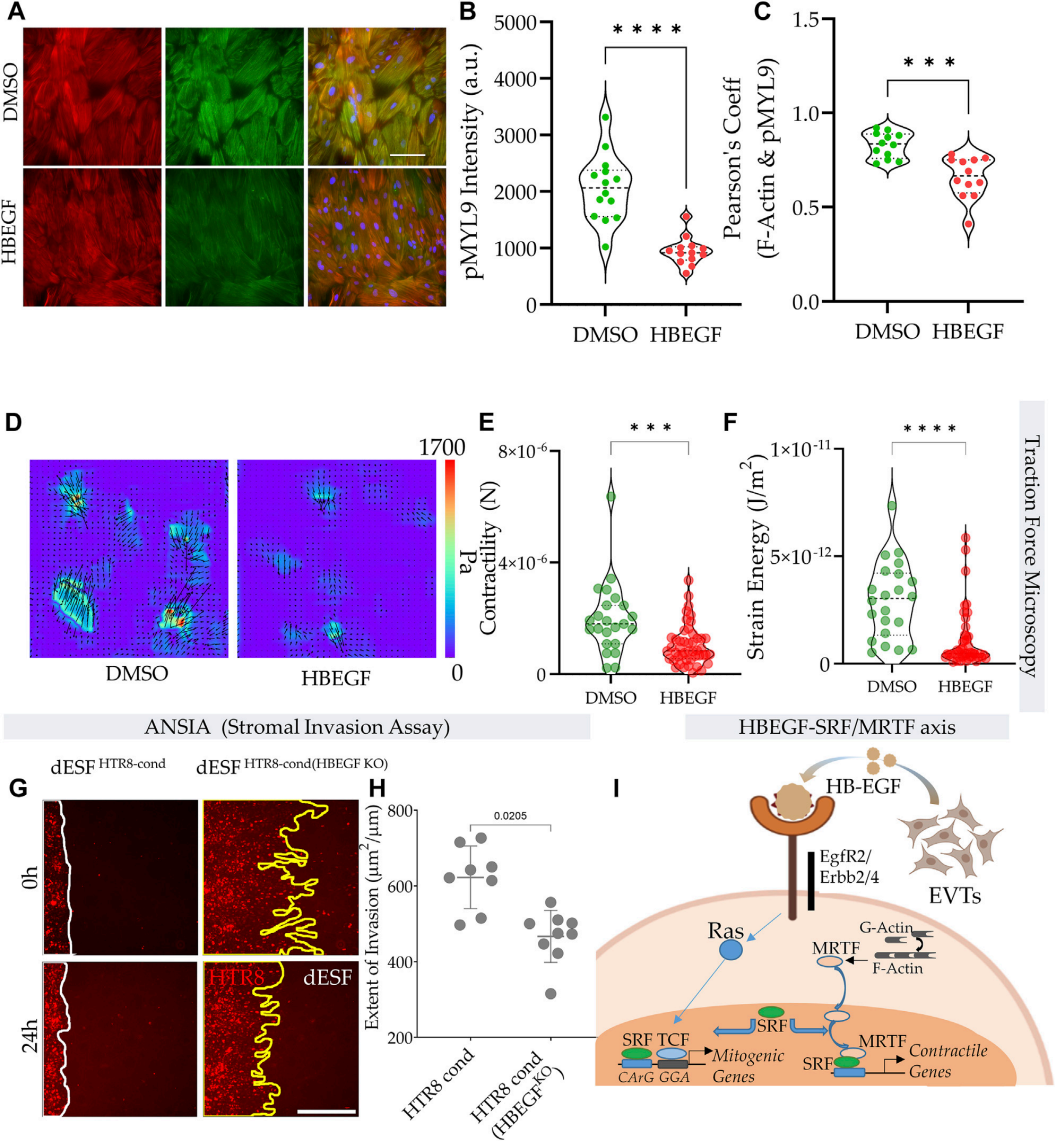
HB-EGF from EVTs reduce contractile force generation in dESFs reducing decidual resistance to invasion. **(A)** Immunofluorescence images of dESFs treated with DMSO control, and HB-EGF (HBEGF) showing F-actin, phospho-MYL9, and combined expression; scale bar = 25 mm. **(B–C)** Average intensity per cell for phospho-MYL9, and Pearson’s coefficient showing F-actin and phospho-MYL9 colocalization. **(D–F)** HB-EGF reduces dESF contractile force generation. **(D)** Stress vector spatial maps showing representative examples of dESFs treated without, or with HB-EGF for 24 h. Individual cell contractility shown in **(E)**, and strain energy density shown in **(F)**; *n* > 20. **(G–H)** HB-EGF decreases dESF resistance to HTR8 invasion. **(G)** Representative images of HTR8-dESF interface at time 0 and 24 h shown for dESFs pre-treated with conditioned medium from wild-type HTR8, or from HTR8 with gene silencing for HB-EGF. **(H)** Quantification of the extent of stromal invasion by HTR8 for conditions in j; *n* > 10. **(I)** Schematic showing the balance of SRF transcriptional association with either TCF co-factors acting through CaRG response element, or with MRTF-A/B co-factors present in nucleus upon release by polymerized actin in the cytosol.

HB-EGF produced by EVTs rewired the SRF axis from SRF-MRTF mediated to likely SRF-TCF mediated gene expression. Earlier results also suggested reduction of SRF-MRTF signaling to decrease contractile force generation. Immunofluorescence in dESFs showed a marked reduction in phospho-MYL9 intensity after treatment with HB-EGF, as well as colocalization with F-Actin (Figures 6A–C). The dramatic reduction in phosphor-MYL9 levels was also matched phenotypically in a profound reduction in contractile force generation in dESF after HB-EGF treatment. Traction force microscopy confirmed that treatment of dESFs with HB-EGF decreased cellular contractility by nearly 3 folds, a remarkable effect on cell mechanics (Figures 6D,E). Strain energy also showed a rapid decrease in dESFs after treatment with recombinant HB-EGF (Figure 6F).

We finally asked if HB-EGF mediated change in MRTF regulated dESF contractility and resistance to invasion. Using ANSIA, we tested the effect of dESF resistance after pre-conditioning with medium from HTR8, wild-type or those with silenced HB-EGF. We found that when dESFs were pre-conditioned with medium from HTR8 with HBEGF gene silencing, their resistance to invasion was significantly increased (Figures 6G,H). Overall these data suggested an intercellular signaling communication between EVTs and dESFs, wherein HB-EGF produced by EVTs rewire the balance of contractile signaling from SRF-MRTF to SRF-TCF transcription in dESFs, reducing mechanical resistance to invasion, thereby facilitating EVT invasion into the maternal stroma.

## Discussion

Collective migration of epithelial cells into the stromal compartment underlines many physiological processes, including gastrulation (Dumortier et al., 2012), wound healing (Li et al., 2013), placentation (Knofler and Pollheimer, 2013), as well as cancer dissemination (Gaggioli, 2008). For long, the invading cells, either trophoblasts in placentation, or cancer cells in metastatic initiation, were considered as the primary agents in stromal invasion, with the stroma considered as a passive barrier to breach (Hanahan and Weinberg, 2000, 2011). Recent decade has increased our appreciation of the role of cancer associated fibroblasts in abetting tumor dissemination, but it is not yet clear whether fibroblasts assist, or resist cancer invasion, as evidence point to either scenario. It is however recognized that cancer cells themselves can prime stromal fibroblasts, incorporating them to facilitate stromal invasion (Ansardamavandi and Tafazzoli-Shadpour, 2021).

Here, we report a mechanism used by the invading extravillous trophoblasts (EVTs) to reduce the decidual resistance to invasion, specifically by decreasing the high contractile forces in decidual endometrial fibroblasts (dESFs) which phenocopy the mechanics of activated fibroblasts. It is notable that extravillous trophoblasts (EVTs) are a recently evolved cell type present in great apes, characterized by excessively deep placental invasion during pregnancy. It would be interesting to test if trophoblasts which are of more ancestral lineage also employ a similar strategy to reduce mechanical resistance by decidual fibroblasts.

A key finding in this report is the metabolic transition of ESFs in response to decidualization and their subsequent interaction with EVTs. Cellular contractility is an intense energetic process requiring high ATP levels to maintain steady state of ATP hydrolysis required for actomyosin cycling (Dos Remedios et al., 2003; Sleep et al., 2005). Harnessing the variance in cellular contractility within dESF populations, we showed that cellular glucose uptake and strain energy were highly correlated. Increase in contractile phenotype in decidualization was also paralleled by a transition towards energy efficient OxPhos pathway for ATP generation. However, concomitant increase in glycolysis, glucose uptake and activation of biosynthetic pathways suggested that other biosynthetic processes associated with decidualization were likely sustained through increased glucose uptake. Increase in OxPhos can also lead to higher reactive oxygen species generation and oxidized cellular state, but enhanced mitochondrial competency and glycolytic flux can keep the reduced cellular state which is necessary to maintain the cellular collagen production (Heid et al., 2017). We found that interaction with EVTs both reduced glycolysis, as well as Oxphos, reducing the energetic state of the dESFs, paralleling the reduction in contractile force generation. A comprehensive metabolic assessment of decidualization has not been presented before, and our data can set the metabolic and mechanical hallmarks to measure decidualization phenotype.

We identified a key paracrine axis recruit decidual fibroblasts as EVT’s partners in invasion. A marked activation of SRF-MRTF transcriptional activity indicated its role in the increased contractile force generation during decidualization of ESFs. Somewhat surprisingly, we found that the effect of SRF-MRTF activation on cellular contractility was achieved not directly via transcription of the fibroblast specific myosin light chain 9 (MYL9) (Zhang et al., 2013), but by its increased phosphorylation. This is likely achieved by increased MRTF induced transcription of the upstream myosin light chain kinase (MLCK) (Johnson et al., 2014).

Because SRF is known to competitively bind either to MRTF, or TCF, a transcriptional co-regulator activated by MAPK signaling (Gau and Roy, 2018), we searched for potential ligands encoding genes expressed in the trophoblasts itself which could potentially activate MAPK signaling in the recipient dESF cells. We found that HTR8 expressed a key secreted ligand for the epidermal growth factor receptor signaling, HBEGF (heparin binding epidermal growth factor like growth factor), which could potentially rebalance the SRF association with MRTF in the nucleus. Decidual ESFs expressed genes encoding receptors which HBEGF could bind to, and pathway analysis suggested a strong activation of MAPK signaling in dESFs after co-culture with HTR8. Other evidence, including immunocytochemistry, also pointed towards a shift in the transcription of genes in dESFs from the initial SRF-MRTF activated genes to the SRF-TCF activated CARGome genes, which contain a consensus CARG sequence in their promoter regions (Sun et al., 2006). Treatment of dESFs with HB-EGF resulted in significant and substantial reduction of cellular contractile forces and mechanical strain energy, highlighting its novel role as a key paracrine mechanoregulator. We also quantitatively tested the effect of HB-EGF on regulating decidual resistance to invasion using ANSIA, a platform we have developed to quantitatively measure stromal invasability as a phenotype with high sensitivity (Novin et al., 2021). These data indicate that HBEGF secreted by EVTs rebalance SRF transcription from the decidual specific MRTF mediated to MAPK driven TCF mediated, resulting in significantly reduced contractile force generation and increasing invasability.

Being a potent activator of the mitogenic MAPK signaling, HB-EGF has been studied primarily in cancers as an oncogenic target, for its role in activating cell proliferation and anchorage-independent growth through autocrine signaling (Ray et al., 2014; Hsieh et al., 2017). HB-EGF autocrine signaling is associated with breast cancer intravasation and metastasis in macrophage independent fashion, and owing to its activity on MAPK signaling, is a potent mitogen in many cancers, including lung (Yotsumoto et al., 2017; Wang et al., 2020), pancreatic (Ray et al., 2014), and breast tumors (Sethuraman et al., 2018). HB-EGF is frequently referred to as an “immediate early gene” owing to its rapid transcription in response to cytoprotective or oncogenic stimuli, including ischemia reperfusion in the heart (Xia et al., 2003), and in tumorogenesis (Mccarthy et al., 1995). Although AP-1 related transcription in response to stretch in smooth muscle cells can induce transcription of HB-EGF (Park et al., 1999) the role of HB-EGF as a modulator of myofibroblast transformation are not well studied. We found that HB-EGF can tilt the balance from a high contractility promoting state in a myofibroblast like decidual fibroblasts to a less mechanically resistive cell, primarily by shifting association of SRF to the MAPK induced TCF signaling. Again, this was achieved by reduced phosphorylation of MYL9 which engages with actin bundles to promote actomyosin contractility. The effect of HB-EGF on dESF mechanics was profound, reducing their contractility and strain energy density by 3 folds, as well as reducing their mechanical resistance to trophoblast invasion. This finding may shed light on previous reports where HB-EGF was found to be a suppressor of liver fibrosis (Huang et al., 2012).

In light of placental invasion and cancer metastasis being parallel models, in so far as the stromal fibroblast response is concerned in regulating the process of invasion, we show that invading cells can use paracrine signals to modulate mechanical forces in the resistive cells, facilitating invasion. As HB-EGF is produced by the invading cells themselves, it presents a therapeutic target to control the transformation of fibroblasts to reduce their mechanical resistance to invasion by cancer, or to modulate placental invasion in pregnancy related disorders.

## Methods

### Cell isolation and maintenance

Human endometrial stromal fibroblasts (hESFs) were isolated from normal patient biopsies obtained by Charles Lockwood Lab at Yale, and obtained from Gil Mor group (Krikun et al., 2004; Graham et al., 1993), as well as from patient biopsies obtained from UCHC Biorepository. HTR8 and BeWo cells were obtained from ATCC. hESF cells were maintained in phenol-red free DMEM/F12 50:50 containing 25 mM glucose, and supplemented with 10% calf serum (charcoal stripped), 1% antibiotic/antimycotic, and ITS (insulin, transferrin, and selenium). hESFs were decidualized with 0.5 mM 8-B-cAMP (Cayman), and 0.5 mM medroxy-progesterone acetate (MPA) for 4 days in DMEM/F12 supplemented with 2% charcoal stripped calf serum.

For co-culture, trophoblasts were labeled with DiI (or stably transduced with plasmid expressing H2B-bound mCherry driven by CMV promoter), washed multiple times with PBS and mixed with decidual ESFs (dESFs) at 1:1 ratio for 3 days. During co-culture cAMP was withdrawn owing to its toxicity to HTR8. For co-culture controls, dESFs were also maintained in decidualization medium without cAMP. Conditioned medium from HTR8 was supplemented with MPA to maintain consistency across all experimental conditions.

### Fluorescence assisted flow sorting

After co-culture, cells were detached from the substrate with 0.25% Trypsin-EDTA, and protease was neutralized with excess medium when all cells were detached. Serum free medium at 4°C was used for cell suspension, and sorting was performed using BD FacsARIA II in UConn Health Flow Core. PE. Cy5 channel was used to separate HTR8, and dESFs using conservative gating (thereby only selecting DiI^lo^ and DiI^hi^ cells, leaving most cells in the middle spectrum to avoid trace potential chance of cell fusion, or dye uptake from apoptotic cells). FACSDiva 6.0. or Flowjo was used for anlaysis. After sorting, cells were directly collected in RNASelect to stabilize RNA, or were collected in 10% FBS containing medium for further experiments. The same procedure was applied on control cells, even though there were only one type of cells in the monoculture dESFs.

### RNA sequencing and transcriptomic analysis

Cells were lysed with buffer RLT, and RNA was isolated using RNeasy Mini Kit (Qiagen) following manufacturer’s instructions. RNA integrity was evaluated with Bioanalyzer 2100 (Agilent) and samples with RIN ∼8 were used for library preparation. Library prep and RNA sequencing were performed by Novogene Inc. NCBI GRCh38 genome assembly were used to align the Reads using HISAT2 pipeline with default parameters. Reads were counted using HTSeq, and DESeq2 was used to estimate the Fold changes and statistical significance (*p*-values) for differential expression. Wald test was used to calculate *p*-values for differential expression and the moderated log2 fold change was used for the differential analysis. To calculate the gene sets (Gene Ontology/KEGG) enriched in the DE genes upon decidualization or HTR8, Fisher exact test was used to calculate the over-representative of terms using hypergeometric test followed by correction for multiple testing (Kolberg et al., 2020). Activation scores of transcription factors and canonical pathways analysis were performed using Ingenuity Pathway Analysis (IPA, Qiagen Inc.). Gene set enrichment analysis (GSEA) (Subramanian et al., 2005) were performed on the genes that are downstream of Mrtf (Gualdrini et al., 2016). Hierarchical clustering was performed using UPGMA method with Euclidian distance on z-scores as mentioned earlier (Afzal et al., 2022). Briefly, a subset of genes was selected for clustering that were differentially expressed in either condition, and are known to be downstream of SRF-MRTF or SRF-TCF axis (Gualdrini et al., 2016; Esnault et al., 2017).

Traction force microscopy: Traction force gels were fabricated using protocols previously described (Colin-York et al., 2017). Briefly, coverslips for gel attachment were cleaned using ethanol as well as sonication, followed by treatment with air plasma, and activated with 0.5% glutaraldehyde and 0.5% (3-Aminopropyl) triethoxysilane. Coverslips to be used in beads coating were treated with air plasma, and were coated with 0.01% poly-L-lysine (PLL) before another coating of carboxylate-modified microspheres (diameters = 0.2 um) (Thermo Fisher). Gel precursor solution containing 7.5% acrylamide and 0.15% bis-acrylamide was degassed for 30 min s and thereafter mixed with 0.1% tetramethylethylenediamine and 0.1% ammonium persulfate, and sandwiched between silane-activated coverslips and bead-coated coverslips for 20 min s. After polymerization of polyacrylamide in between the coverslips, bead-coated coverslips were peeled, and the resulting traction force gels coated with 50 ug/ml collagen type I using sulfo-SANPAH (Thermo Fisher) overnight at 4°C. UV was used to sterilize gels for at least 2 h before cell seeding. Images containing microbeads location before and after cells trypsinization were recorded using Zeiss Observer A1 microscope. Traction forces were calculated using protocols previously described (Bauer et al., 2021).

Measurement of oxidative phosphorylation and glycolysis: XF analyzer from Agilent Technologies was used to monitor cellular energetics (Reid et al., 2013; Afzal et al., 2017; Afzal et al., 2022). Cells were cultured in 96 wells XF plate and the assay was repeated three times to estimate the energetics of cells. Oxygen consumption rate (OCR) which reflect the rate of change of dissolved O_2_ in each well was used to estimate OxPhos and change in extracellular acidification rate which is measured as a change in extracellular pH change was used to estimate glycolysis. Basal rates reflect respiration or pH in the absence of added compounds or metabolic inhibitors. Oligomycin (4 µM) was used to inhibit mitochondrial F1Fo-ATP synthase, rotenone (2 µM) was used to inhibit Complex 1 of ETC, antimycin A (2 µM) was used to inhibit complex 3 of ETC, FCCP (500 nM) was used to uncouple mitochondria to estimate maximum respiratory capacity, and iodoacetate (100 µM) was used to inhibit glycolysis (glyceraldehyde-3-phosphate dehydrogenase). Fresh compounds were prepared and dissolved in the XF assay media immediately before the experiment.

Respiration fractions were calculated as follows (Pesta and Gnaiger, 2012; Afzal et al., 2017).

1) *Coupled respiration*: OCR sensitive to oligomycin inhibition which represents OCR used for mitochondrial phosphorylation of ADP.
2) *Uncoupled respiration*: Increase in OCR after FCCP addition which reflects maximal oxygen consumption capacity of mitochondria.
3) *Total mitochondrial respiration*: OCR fraction sensitive to inhibition by rotenone + antimycin.
4) *Glycolytic fraction*: ECAR fraction sensitive to iodoacetate.

OCR/ECAR ratios were used to estimate relative contribution of OxPhos versus glycolysis to cellular energetics as it is independent of cell number. To normalize the respiratory rates, we lysed the cells after the XF assay and quantified the cell number using Picogreen DNA assay (ThermoFisher Scientific) following manufacturer’s instructions.

Cellular ATP quantification: ATP was measured using the ATP Determination Kit (A22066, ThermoFisher scientific) using previously published protocols (Afzal et al., 2017). Reaction solution was prepared for 100 µL reaction volume per well. Cells were lysed using passive lysis buffer (Cat. #E1941, Promega) in cultured well for 15 min, and a reaction solution was added in each well and signal was immediately monitored using luminometer. Standard curves were used to estimate the amount of ATP/well and ATP signal was normalized to cell number using the Picogreen DNA assay (ThermoFisher Scientific). Following ATP measurements were performed using inhibition of metabolic pathways in the cultured cells just before the ATP assays as mentioned in detail (Afzal et al., 2017).

Oligo-sensitive ATP %: ATP levels that were sensitive to the inhibition of ATP synthase using oligomycin (4 µM) for 30 min on live cultured cells. This ATP fraction reflects the ATP produced by cells when OxPhos is inhibited. Ox-Phos ATP percentage is calculated as follows: Oligo-sensitive ATP/basal ATP × 100 in each of three experiments.

Fluorescence microscopy: Invasion assay, as well as immunofluorescence imaging was performed on Zeiss AxioObserver, Z1 microscope with either 10 × or ×20 objectives (EC Plan-Neofluar ×10/0.3, and Plan-Apochromat 20x/0.8 M27) and Hamamatsu ORCA Flash camera. The light source used was SOLA light engine, and image acquisition was performed using Zeiss Zen 2.6 software.

### Gene silencing

Gene knockout was achieved by using pre-prepared synthetic sgRNA (IDT). hESF (or HTR8) were transfected with sgRNA and recombinant Cas9 (IDT) using CRISPRmax reagent (Invitrogen). Specifically, a cocktail was created by mixing 1) solution1: 24 µL OPTIMEM and 1 uL CRISPRMAX, and 2) solution 2: 10 nmol gRNA, 15 nmol Cas9, 1.5 µL CRISPRMAX Plus reagent and remaining OptiMEM to make a 30 µL solution. Solution 1 and 2 were mixed and incubated for 15 min before being drop dispensed for a 24 well containing hESF, dESF, or HTR8 cells at 70%–80% confluency in culture medium with no serum. Cells were used after 48 h of transfection, and observation completed within 48 h thereafter.

### Immunoblot

Cells were lysed in RIPA lysis buffer (Cell Signaling Technology 9806) containing protease inhibitors (Sigma-Aldrich P8340). BCA kit (Thermo Fisher Scientific) was used for protein quantification. 20 ug denatured proteins (95°C for 2 min in SDS) were loaded on 4%–12% NuPAGE Bis-Tris Gel (Thermo Fisher Scientific NP0322BOX), transferred to polyvinylidene difluoride (PVDF) membranes and blocked with 5% BSA for 1 h at room temperature. Antibodies used were: anti-MKL1 (Cusabio G0615A), MYL9 (Proteintech, Inc. 29504-1-AP), phosphor-MYL9 (Proteintech, Inc. 15354-1-AP). Primary antibodies were incubated overnight at 4°C, followed by incubation with GAPDH (Cell Signaling Technology 5174) for 1 h at room temperature. Subsequently, samples were incubated with HRP-linked anti-rabbit secondary antibody (GE healthcare NA9340) for 1 h at room temperature. An enhanced chemiluminescence reagent (Thermo Fisher Scientific 34095) was used to visualize the bands.

### Fabrication of ANSIA platform

ANSIA platform was fabricated by methods previously described (Novin et al., 2021). Briefly, nanotextured substrate was fabricated using pre-fabricated molds, or created using photoresist spun on silicon wafers, and patterned using electron-beam lithography. After developing the photoresist, the exposed silicon was etched with a deep-reactive ion etcher, allowing for the formation of submicron parallel ridges. Residual photoresist was removed using ashing, and diced into silica master for replica molding. Polyurethane was drop-dispensed onto the silicon master and thereafter pressed with a polyethylene terephthalate (PET) film, and subsequently cured with UV (λ= 200–400 nm, 100 mJ/cm^2^) for 1 min. After peeling the mold, the revealed patterned PUA was overcured overnight with UV to terminate residual acrylate groups. Patterns were also purchased from Nanobiosurface for some experiments.

The PET-PUA mold was employed as a replica mold to transfer nanotopographic pattern on glass substrate using capillary force lithography (CFL). Glass coverslip was cleaned using NaOH (0.1 M for 1 h), washed with DIH_2_O, and dried. Propylene glycol monomethyl ether acetate and phosphoric acrylate were mixed in a ratio of 10:1 as a primer and spin coated on the coverslip. The coverslip was baked for 40 min at 68°C. After dispensing PUA precursor dropwise on the primed coverslip, the mold was placed reversibly, and cured in UV (λ= 250–400 nm, 100 mJ/cm^2^) for 1 min, peeled, and overcured in UV overnight.

Fabricated or procured substrates were then seeded with cells in the described pattern to create well defined juxtaposed interfaces between fluorescently labeled trophoblasts, and unlabeled stromal fibroblasts using stencils. A stereolithographic plastic mold was used to create polydimethylsiloxane (PDMS) stencil. The stencil was casted in the mold by mixing monomer and cross-linker in a 1:10 ratio, degassing, and curing at 80°C for 4 h. Topographic substrates were coated with 0.1% w/v collagen type I overnight after corona treatment for 30 s. PDMS stencil was placed, while keeping the setup in a vacuum chamber to allow air under the stencil to escape. H2B-mCherry expressing HTR8 were seeded on the stencil at a density of 10^7^ cells/ml, allowed to attach and form a monolayer in 8 h. Unattached cells were washed out 3 times with PBS, the stencil removed to reveal a cleared area. This cleared area was then seeded with stromal fibroblasts at a typical density of 5 × 10^7^ cells/ml. Unattached cells were washed after 4–6 h of seeding, and live cell microscopy performed immediately in a basal medium consisting of 1:1 ratio of HTR8 and ESF medium without cAMP. Any additional factors were added to the medium according to experimental requirement.

Analysis of stromal invasion & determination of invasive parameters: For each time stamp, images were converted to binary using OTSU thresholding technique (Otsu, 1979). Images were dilated employing a 2 × 4 kernel iteratively ∼15 times. Remaining black interspersed holes were thereafter closed using a trapezoidal 9 × 9 kernel applied in 40 iterations. Dilation and closing resulted in an almost completely whitened mask, which marked the region occupied by invading trophoblasts, and another almost completely black region occupied by stromal fibroblasts, also containing interspersed white regions occupied by the escaped trophoblasts. Approximating a polygon to the contour of the largest connected segment then provided the final invasion region. The escaped cells outside this region were isolated using watershed segmentation (Najman and Schmitt, 1994). By identifying the convex vertices of the polygon, the invasive forks were marked, and the extent of invasion was determined by calculating the area occupied by the main region of the trophoblasts for each time point. For each invasive fork, we then calculated the horizontal distance it travelled starting from its initial positon.

### Statistical analysis

Statistical analysis was performed using students *t*-test unless otherwise mentioned with each result. Data is presented as ± SD, or ±SEM as mentioned in each results section and only significant *pvalues* are reported with each test.

## Data Availability

The data presented in the study is deposited in the NCBI’s Gene Expression Omnibus repository, and is accessible through GEO Series accession number: GSE197810 (use token: kripqmwozfitliv).
